# A New Technique for Simple Renal Cyst: Cystoretroperitoneal Shunt

**DOI:** 10.1155/2009/906013

**Published:** 2009-07-09

**Authors:** Onder Canguven, Cemal Goktas, Faruk Yencilek, Cihangir Cetinel, Selami Albayrak

**Affiliations:** ^1^Urology II Clinics, Kartal Training and Research Hospital, 34738 Istanbul, Turkey; ^2^Urology Department, Yeditepe University, 34758 Istanbul, Turkey

## Abstract

*Purpose*. To evaluate the results of patient symptoms and radiologic outcomes of cystoretroperitoneal shunt (CRS) technique in the treatment of symptomatic simple renal cysts. *Patients and Methods*. In a prospective study, 37 patients with a simple renal cyst were treated with ultrasound-guided percutaneous CRS-catheter. Radiological success was indicated as no recurrence of the cyst or a reduction in cyst volume by at least half. *Results*. CRS technique was performed successfully in 36 patients with a simple renal cyst. The mean size of all cysts decreased from 8.8 cm (range 7 to 14) to 1.7 cm (range 0 to 9; *P* < .001). Symptomatic success (pain relief) was achieved in 29/36 (80.5%) of patients, and radiographic success was achieved in 23/36 (63.8%) of patients, with a median follow-up of 16 months (range 6 to 24). *Conclusion*. Ultrasound-guided percutaneous CRS technique for simple renal cysts is fast, safe, effective, and inexpensive.

## 1. Introduction

Simple renal cysts are common, with incidence increasing with age from 0.22–0.55% in children to >5% in the fourth decade, and up to 36% in the eighth decade of life [1]. The Bosniak renal cyst classification was first introduced in 1986 and has been accepted by urologists and radiologists as a way of diagnosing, discussing, and determining the management approach to cystic renal masses [2]. The vast majority of renal cysts are simple; they are thin and smooth-walled, with no calcification, septation or enhancement after contrast studies, corresponding to Bosniak category I [2].

Although simple renal cysts are usually asymptomatic, flank pain is the most common symptom. A palpable flank mass, hematuria, hypertension, and compression of the pelvicaliceal system also can occur [3]. Pain, infection, and obstructive uropathy are the major indicators for surgical intervention [3]. Operations can be performed by open surgery, percutaneous aspiration, with or without sclerosis, and, most recently, laparoscopic surgery [1, 3]. 

Open surgical cyst decortication for pain relief through an abdominal incision is associated with considerable morbidity and protracted convalescence [3]. Percutaneous aspiration presents minimally invasive management but, because of frequent relapses, sclerotherapy is advised after cyst aspiration [1]. Treatment with the sclerosing agents varies significantly among studies with respect to the time of exposure to agents, concentration and volume, and number of sclerotherapy sessions [1, 4, 5]. Laparoscopic decortication of cysts is less invasive than open surgery; however, the procedure is expensive and both procedures must be performed under general anesthesia [1]. 

Open surgical or laparoscopic decortication of cysts creates a window between the renal cyst and retroperitoneal area. We designed a new technique in order to achieve the same goal and eliminate the risks associated with both surgeries. In this study, we describe and analyze the feasibility, safety, and efficancy of “cystoretroperitoneal shunt (CRS) technique” in simple renal cysts.

## 2. Patients and Methods

From April 2004 through February 2008, 37 patients underwent the CRS technique for symptomatic simple renal cysts. The study was approved by the local ethics committee. All patients signed a detailed consent form that listed details about the procedure and possible complications. All cysts were unilateral, single and in Bosniak category I [2]. Complex cysts, pregnant patients, and patients with solitary kidney were not included in this study.

### 2.1. Measurements and Follow-Up

All patients were evaluated in the urology clinic, where the cysts were localized and characterized by ultrasonography. Additionally, computed tomography was performed for Bosniak classification. The volume of the cysts was calculated with an ellipsoid formula, whereby the product of the three orthogonal diameters was multiplied by 0.52 (volume = height × width × length × 0.52). The volume of the cyst was compared to that estimated before treatment and represented as a percentage of the initial value. Radiological success was designated as no recurrence of the cyst or a reduction in pretreatment cyst volume by at least half. The following studies were also performed at enrollment: after 7 days, and after 1, 3, 6, 9, 12, 18, and 24 months, clinical examination and laboratory tests that included urinalysis, serum creatinine level, and coagulation profile.

Flank pain and mass, hypertension, and hematuria were documented before treatment. The subjects were considered to have hypertension based on data obtained from their histories and if the values of their systolic/diastolic blood pressure were 140/90 mm Hg or greater. The blood pressure measurement was conducted on the day before the procedure and at each examination during the follow-up period. The patients were also asked if they did or did not have pain. The severity of pain was not evaluated.

### 2.2. Technique

**Figure 1:**
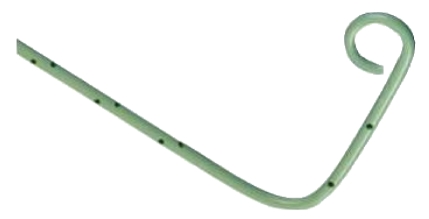
Seven French ×50 cm polyethylene drainage ring catheter.

**Figure 2:**
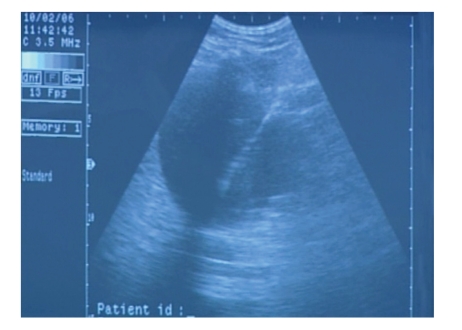
CRS-catheter is introduced until reached the opposite wall of the cyst cavity under ultrasound guidance.

**Figure 3:**
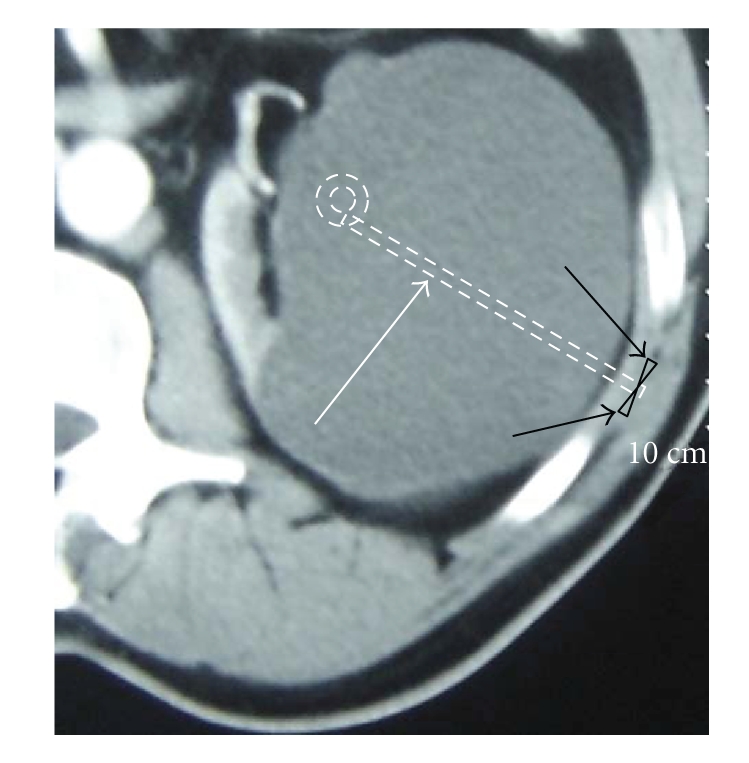
Schematization of CRS technique. *Black arrows: * sutures at two different sites. *White arrow:* CRS-catheter after the introducer needle taken out.

The CRS technique was performed with a single step 7 French ×50 cm drainage ring catheter (Angiotech, PBN Medicals, Denmark) (Figure 1). The catheter had 32 side holes and hydrophilic coating for minimal friction during insertion. With the patient in a flank position for a retroperitoneal approach and after determination of the puncture site, antiseptic preparation and local anesthesia with 2% lidocaine hydrochloride (1 mg/kg) was given before a small puncture wound was made on the skin. The CRS-catheter was inserted until it reached the opposite wall of the cyst cavity under ultrasound guidance (Figure 2). As much cystic fluid as possible was aspirated through the catheter, and the volume of aspirated fluid was recorded. A fluid sample was sent for bacteriologic and cytologic examinations. In order to secure it, the catheter was sutured with a nonabsorbable material (4–0 Nylon or Prolene suture) at two different sites subcutaneously (approximately 0.5 cm below the skin surface, as shown in Figure 3). After suturing subcutaneously, the CRS-catheter was cut above the sutures and just below the skin. The skin, which was about 1 cm, was closed with the same nonabsorbable suture material, which was removed after 7 days. After the procedure, the patient rested in bed for 2 hours and was discharged from the hospital on the same day. We removed the CRS-catheter after 3 months with a small incision made on the introduced area, the sutures cut, and the catheter easily removed.

### 2.3. Statistical Analysis

Statistical analyses were performed with GraphPad Prism 4 software for Windows. Student's *t*, Mann Whitney and Fisher's exact tests were done. A value of *P* < .05 was considered significant.

## 3. Results

**Figure 4:**
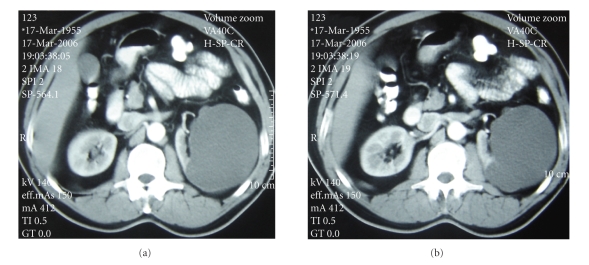
Left simple renal cyst before treatment.

**Figure 5:**
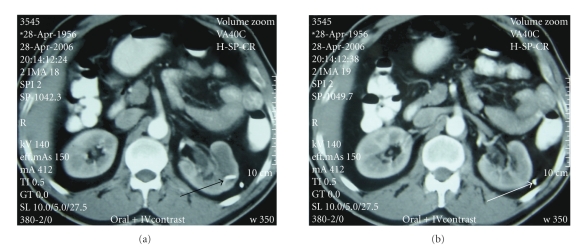
Six weeks after treated with CRS-technique. *Black arrow: * renal cyst component of CRS-catheter. *White arrow:* retroperitoneal component of CRS-catheter.

The subjects were 15 men and 22 women with a median age of 48.5 years (36–65 years) (Table 1). The median operative time (from skin incision to placement of final dressing) was 29 minutes (range 22 to 46). The mean size of all cysts decreased from 8.8 cm (range 7 to 14) to 1.7 cm (range 0 to 9; *P* < .001). The mean volume of the cysts was 354.8 ± 38.3 mL (range, 170–924 mL). The cysts volume dropped less than 50 mL in volume in all patients (100%) in 24 hours after the CRS-catheter insertion. During follow-up period, one (2.7%) of the cysts recurred shortly after disappearance. We excluded that case from our study group when the catheter was found out of the cyst. The disappearance of the cyst was sustained in other patients 36/37 (97.3%) until the CRS-catheters were removed (Figures 4  and 5). Flank pain was present to variable degrees in all patients before treatment. The flank pain later subsided in all patients whether resolution of the cyst was complete or partial. Symptomatic success (pain relief) after removing of the CRS-catheter was accomplished in 29/36 (80.5%) of patients, and radiological success was gained in 23/36 (63.8%) of patients, with a median follow-up of 16 months (range 3 to 24). The radiological success probability of giant cysts (>350 mL) differed significantly from smaller cysts (<350 mL; *P* < .05). The mean volume of the recurrent cysts was 180.5 ± 73.5 mL (range, 107–264 mL). Only patients with giant cysts underwent further treatment with percutaneous aspiration with ethanol injection. Only seven patients (19.5%) with a giant cyst and persistent pain underwent further treatment with percutaneous aspiration with ethanol injection. Aspiration with ethanol injection alleviated pain in all the patients. Four patients had no pain and three reported decrease in its severity. 

For the 13 patients with associated hypertension, six (46.1%) had well-controlled blood pressure with no medication after the decrease in cyst size. One patient had a wound site infection three days after insertion of the catheter, which was resolved by oral antibiotherapy (ciprofloxacin 500 mg oral tablet, two times a day). Microbiologic and cytologic investigations revealed normal findings.

## 4. Discussion

This study reports on a new CRS-technique used with 37 patients and a median follow-up of 16 months. Radiological success was achieved after catheter removal in 63.8% of patients. Flank pain was present to variable degrees in all patients before treatment. Ultrasonography and computed tomography were used both for diagnosis of renal cyst and to rule out nonrenal etiology for pain. Following treatment, the flank pain subsided in all patients whether resolution of the cyst was complete or partial. Symptomatic success (pain relief) was accomplished in 80.5% of patients after removing the CRS-catheter. Laparoscopic decortication is effective and some authors recommended it as the next step for a recurrence after simple aspiration that initially relieved the pain [1]. We did not consider laparoscopic decortication after failure of CRS-catheter treatment due to possible fibrotic changes in the retroperitoneal area and decided to perform aspiration with ethanol injection. 

Hypertension associated with simple renal cysts is likely to disappear after treatment. In our study, six hypertensive patients (46.1%) benefited from treatment with normalization of blood pressure. Similarly, Touloupidis et al. found that, of 61 patients with hypertension, 29 had improved blood pressures after sclerotherapy of simple renal cysts [6]. 

Renal cysts are common in the adult population [1]. With a mean follow-up 9.9 years, Terada et al. demonstrated that the average rate of cyst enlargement was 3.9% per year [7]. Treatment is indicated in patients with flank or abdominal pain or complications, such as collecting-system obstruction or hypertension. Symptomatic renal cysts have conventionally been treated by percutaneous aspiration with or without injection of sclerosing agents; however, this has a high rate of recurrence [4, 5]. The recurrence rate of simple aspiration with or without sclerosing agent varies between 41%–78% [8, 9] and 32%–100% [10, 11], respectively. Among these various sclerosing agents, ethanol has been widely used since the initial report by Bean [8]. However, the leakage of alcohol from the cyst results in necrosis of the surrounding tissue. Moreover, the systemic diffusion of alcohol may induce central nervous system depression [5]. Several sclerosing agents (e.g., sodium tetradecyl sulfate and povidone-iodine) have been used to diminish side effects and increase success rates [5, 12–14]. Demir et al. demonstrated that ethanol and sodium tetradecyl sulfate are well-tolerated sclerosants for the treatment of simple renal cysts [5]. However, they preferred the latter agent as a first choice because it causes less pain. Madeb et al. showed that povidone-iodine sclerotherapy was followed by a high rate of recurrence and, therefore, not indicated for the treatment of symptomatic simple renal cysts [12].

Laparoscopic decortication of symptomatic renal cysts has been used since the early 1990s [15, 16]. Although the laparoscopic approach to simple renal cysts is effective, technical demands and costs currently limit widespread application. Various investigators have reported high success rates for laparoscopic interventions [3, 17]. However, Shiraishi et al. showed that radiological failure could be seen in up to 19% of patients in the long-term (mean follow-up of 67.2 months), which was rather disappointing compared to the short-term results [15]. 

Previous studies suggested that cyst recurrence after sclerotherapy was a result of inadequate sclerosis of the cyst wall epithelial cells, which continued to produce fluid into the cyst [1]. The secretory activity of the residual cyst wall after decortication is also contributory in cystic recurrence. The needle puncture site for sclerotherapy appears to close soon afterward and does not provide drainage of the cyst. In our technique, we wanted to leave the puncture site open longer. Therefore, the shunt would continue to carry cystic fluid into the retroperitoneal area. We achieved success to some degree with symptomatic relief for the patients. Another explanation might be the fibrotic action inside the cyst wall. Fibrosis might have decreased the cyst to a smaller size, especially in giant cysts. Since the cyst shapes of the treated patients were spherical and smaller rather than aspheric, we believe that a permanent window was created after removal of the CRS-catheter. 

 As in other studies, there was an obvious difference between radiologic and symptomatic success in our study [3, 15]. All studies revealed that the remaining cyst, irrespective of its size, was not always associated with symptoms. Although the success rate after removal of the catheter was similar to other reported success rates, CRS could be an innovative technique with lifelong biocompatible material. Moreover, the CRS technique can easily be done under local anesthesia in an outpatient clinic. 

The major limitation of our study was the lack of a control group for comparison of our results. We might have randomly assigned patients into two groups [4, 5] in a prospective controlled design. However, we could compare our results to those of previous studies. In the future, we plan to carry out this technique with biocompatible catheters. By that time, we will also have obtained the long-term results of the patient group.

In conclusion, the CRS-technique is minimally invasive, well-tolerated, and easily administered under ultrasound guidance and does not require hospital admission. It can be offered to patients who may prefer to avoid risks associated with sclerosing agents and general anesthesia. As for every novel technique, further larger and controlled studies are needed to evaluate the value of the CRS-technique in the treatment of symptomatic simple renal cysts.

## Figures and Tables

**Table 1:** Patient characteristics and preoperative parameter.

Patients (n)	37
Male/female (n)	15/22

Age (yr)	
* *Mean	48.5
* *Range	36–65

Side (n)	
* *Right	12
* *Left	25

Cyst size (cm)	
* *Mean	8.8
* *Range	6–14

Previous cyst aspirations (%)	5 (13.8%)
